# *agtools*: a software framework to manipulate assembly graphs

**DOI:** 10.1093/bioadv/vbag126

**Published:** 2026-05-05

**Authors:** Vijini Mallawaarachchi, George Bouras, Ryan R Wick, Susanna R Grigson, Bhavya Papudeshi, Robert A Edwards

**Affiliations:** Flinders Accelerator for Microbiome Exploration, Flinders University, Bedford Park, SA 5042, Australia; School of Medicine, College of Health, Adelaide University, Adelaide, SA 5005, Australia; The Department of Surgery—Otolaryngology Head and Neck Surgery, Central Adelaide Local Health Network, Adelaide, SA 5005, Australia; Department of Microbiology and Immunology, The University of Melbourne at the Peter Doherty Institute for Infection and Immunity, Melbourne, VIC 3000, Australia; Centre for Pathogen Genomics, The University of Melbourne, Parkville, VIC 3010, Australia; Flinders Accelerator for Microbiome Exploration, Flinders University, Bedford Park, SA 5042, Australia; DOE Joint Genome Institute, Lawrence Berkeley National Laboratory, Berkeley, CA 94720, United States; Flinders Accelerator for Microbiome Exploration, Flinders University, Bedford Park, SA 5042, Australia; Department of Fundamental Microbiology, University of Lausanne, Lausanne CH-1005, Switzerland; Flinders Accelerator for Microbiome Exploration, Flinders University, Bedford Park, SA 5042, Australia

## Abstract

**Motivation:**

Assembly graphs are a fundamental data structure used by genome and metagenome assemblers to represent sequences and their overlap information, facilitating the assembler in constructing longer genomic fragments. Apart from their core use in assemblers, assembly graphs have become increasingly important in a range of downstream applications such as metagenomic binning, plasmid detection, viral genome resolution, and haplotype phasing. However, there is a need for a comprehensive tool that allows programmatic access to manipulate assembly graphs (e.g. parse, convert, filter, and analyze) across different assembly graph formats.

**Results:**

Here we present *agtools*, an open-source Python framework to manipulate assembly graphs produced by commonly used assemblers. *agtools* provides a command-line interface for tasks such as assembly graph format conversion, segment filtering, and component extraction. It also exposes a Python package interface to load, query, and analyze assembly graphs from popular genome and metagenome assemblers. This enables streamlined assembly-graph-based analyses that can be integrated into other bioinformatics software and workflows.

**Availability and implementation:**

The source code of *agtools* is hosted on GitHub at https://github.com/Vini2/agtools and the documentation is available at https://agtools.readthedocs.io/. *agtools* can also be installed from Bioconda (https://anaconda.org/bioconda/agtools) and PyPI (https://pypi.org/project/agtools/).

## 1 Introduction

Assembly is the process of reconstructing genomes from sequencing reads and is a crucial step in many genomic and metagenomic analysis workflows. Modern assemblers typically use graph structures, collectively referred to as *assembly graphs*, to represent sequences and their connections. Assembly graphs are generated as intermediate outputs from assemblers in various formats. For example, SPAdes (Bankevich *et al*. 2012), Flye (Kolmogorov *et al*. 2019), hifiasm-meta (Feng *et al*. 2022), and myloasm (Shaw *et al*. 2026) use the Graphical Fragment Assembly (GFA) format (GFA Format Specification Working Group 2014), MEGAHIT (Li *et al*. 2015) and early versions of SPAdes (prior to version 3.10.0) use the FASTG format, and the String Graph Assembler (SGA) (Simpson and Durbin 2012) uses the ASQG format. Despite the differences in format, vertices (or nodes) represent sequences (or segments) and edges represent connections or overlaps (or links) between the sequences (Pevzner *et al*. 2001, Myers 2005, GFA Format Specification Working Group 2014, Nurk *et al*. 2017). Formats such as GFA represent more complex information, including *paths* and *walks* consisting of multiple sequences (https://gfa-spec.github.io/GFA-spec/GFA1.html). Terminology also varies somewhat across assemblers: non-branching paths in the assembly graph are often referred to as *unitigs* (Kececioglu and Myers 1995), and longer, optimized paths are resolved into contiguous sequences known as *contigs* (Bankevich *et al*. 2012). Contigs can be further combined into *scaffolds*, which are longer sequences that potentially include N bases to fill gaps (Silva *et al*. 2013, Wick *et al*. 2017). Importantly, terminology and graph definitions are not universally fixed. Different assemblers may adopt slightly different conventions for constructing graphs and representing sequences within them.

Beyond their role in genome and metagenome assembly, assembly graphs are used in many graph-based approaches for downstream applications. In metagenomic binning, assembly graphs are used to group contigs into bins that represent different taxonomic groups based on their local connectivity (Lamurias *et al*. 2022, Mallawaarachchi and Lin 2022a, 2022b, Xue *et al*. 2022, Feng and Li 2024, Mallawaarachchi *et al*. 2024, Xue *et al*. 2024). In population genomics, assembly graphs are used for haplotype phasing, as they represent alternative paths corresponding to different allelic sequences (Garg *et al*. 2018, Henglin *et al*. 2024, Kazantseva *et al*. 2024). In viromics, assembly graphs are used to resolve strains or variants of eukaryotic viruses and bacteriophages (Luo and Lin 2023, Mallawaarachchi *et al*. 2023, Papudeshi *et al*. 2025). Moreover, plasmids can be detected from assembly graphs as isolated circular sequences or distinct subgraphs (Rozov *et al*. 2017, Wickramarachchi and Lin 2022, Bouras *et al*. 2023, Paganini *et al*. 2024). In metagenomic sequence classification, assembly graphs are used to improve classification results based on neighborhood information (Pu and Shamir 2022, 2024). These diverse applications depict the significance of assembly graphs, not just as an intermediate output from assembly, but as a valuable source of biological data and sequence connectivity.

Despite the growing interest in using assembly graphs for downstream applications, there are a limited number of tools that can capture assembler-specific graph structures while allowing users to manipulate them. Table 1 summarizes the capabilities of existing tools based on official documentation and repository implementations, with respect to interfaces, format support, and graph abstraction. Many existing tools can handle only one format and do not allow conversion between different formats. For example, tools such as gfatools (https://github.com/lh3/gfatools), PyGFA (https://github.com/pmelsted/pyGFA), RGFA (Gonnella and Kurtz 2016), GFA for Ruby (https://github.com/lmrodriguezr/gfa), GFAKluge (Dawson and Durbin 2019), GfaPy (Gonnella and Kurtz 2017), and Gfastats (Formenti *et al*. 2022) can only parse and manipulate files in the GFA format, while Pyfastg (https://github.com/fedarko/pyfastg) can only parse files in the FASTG format. Assemblers such as ABySS (Simpson *et al*. 2009) include format conversion tools, but these are often assembler-specific and require the installation of the full software. Moreover, there is no programmatic interface that can comprehensively represent and manipulate different assembly graph structures while integrating assembler-specific information (e.g. contig sequences and paths) to construct higher-level representations such as contig graphs from popular assemblers such as SPAdes and Flye. This creates challenges for researchers who want to incorporate assembly graphs into their workflows, often requiring them to write custom code to perform graph-based analyses. Hence, there is a clear need for an extensible software framework that provides a standardized interface for parsing, manipulating, and analyzing assembly graphs across different assemblers.

**Table 1:** Feature comparison of existing tools with *agtools*.

Tool	Programming language	API	CLI	Graph format^a^ conversion to/from GFA	Export to other non-GFA formats^b^	Integrates assembler-specific contig/path information	Link to software repository
gfatools	C	×	✓	Limited	✓	×	https://github.com/lh3/gfatools
PyGFA	Python	✓	×	×	Limited	Limited	https://github.com/pmelsted/pyGFA
GFA for Ruby	Ruby	✓	×	×	×	×	https://github.com/lmrodriguezr/gfa
RGFA	Ruby	✓	✓	×	×	×	https://github.com/ggonnella/RGFA
GFAKluge	C++	✓	✓	Limited	×	×	https://github.com/edawson/gfakluge
GfaPy	Python	✓	×	Limited	×	×	https://github.com/ggonnella/gfapy
Pyfastg	Python	✓	×	×	×	Limited	https://github.com/fedarko/pyfastg
Gfastats	C++	×	✓	Limited	Limited	Limited	https://github.com/vgl-hub/gfastats
*agtools*	Python	✓	✓	✓	✓	✓	https://github.com/Vini2/agtools

a Convert other assembly graph formats such as FASTG and ASQG to and from GFA format.

b Export GFA files to other non-GFA formats such as the assembly graph into DOT and adjacency matrix formats and the segment sequences into FASTA format.

✓ indicates that the feature is explicitly supported and documented by the tool, × indicates that the feature is not supported, and “Limited” indicates that the feature supports limited functionality.

API: application programming interface; CLI: command-line interface.

In this article, we introduce *agtools*, an open-source Python framework designed to analyze and manipulate assembly graphs. In contrast to tools that focus primarily on file parsing or format conversion, *agtools* provides a standardized graph abstraction layer, supports the parsing of assembler-specific graphs and relevant graph representations, and exposes both a command-line and a Python package interface for downstream analysis (Table 1). These design choices facilitate the integration of *agtools* into larger bioinformatic workflows that require assembly-graph-based operations in a modular and reproducible manner.

## 2 Implementation and features

### 2.1 Definitions

In this work, we explicitly distinguish between two assembly graph representations. A *unitig graph* refers to an assembly graph in which nodes correspond to unitigs (i.e. maximal non-branching paths) (Myers *et al*. 2000, Li 2012), whereas a *contig graph* refers to a higher-level graph in which nodes correspond to contigs and edges represent relationships inferred from their paths in the unitig graph. While some assemblers provide both unitig- and contig-level information within the same assembly graph file (e.g. SPAdes and Flye), others provide only the contigs in the final assembly graph file (e.g. MEGAHIT and myloasm). To ensure consistency across assemblers and to support diverse assembly graph representations, we treat unitig and contig graphs as distinct graph representations.

### 2.2 Implementation

*agtools* is implemented in the Python programming language and runs on the Linux and macOS operating systems. It is designed as a software framework rather than as a standalone analysis tool, with an emphasis on modularity, extensibility, and interoperability with existing bioinformatics software. To support these goals, *agtools* provides a command-line interface (CLI) with various subcommands to query and manipulate assembly graphs, and an application programming interface (API) to represent assembly graphs for downstream analyses. *agtools* includes over 200 test cases covering its CLI and API functionality.

Unitig- and contig-level assembly graphs are represented using python-igraph (Csárdi and Nepusz 2006), and sequences are represented using Biopython (Cock *et al*. 2009). This design allows *agtools* to leverage established and well-optimized libraries for graph manipulation and sequence handling while maintaining a consistent internal representation across different assembly graph representations.

*agtools* supports multiple assembly graph formats (e.g. FASTG and ASQG via conversion to GFA) and, with respect to GFA specifications, supports only the GFA 1 format (https://gfa-spec.github.io/GFA-spec/GFA1.html), which is used by many assemblers. Support for GFA 1 was a deliberate design choice, as it provides stable and consistent graph representations across modern assemblers. Although a GFA 2 specification (https://gfa-spec.github.io/GFA-spec/GFA2.html) has been proposed, it has not been widely adopted by assemblers and is therefore currently not supported.

### 2.3 Modular architecture of *agtools*

**Figure 1:**
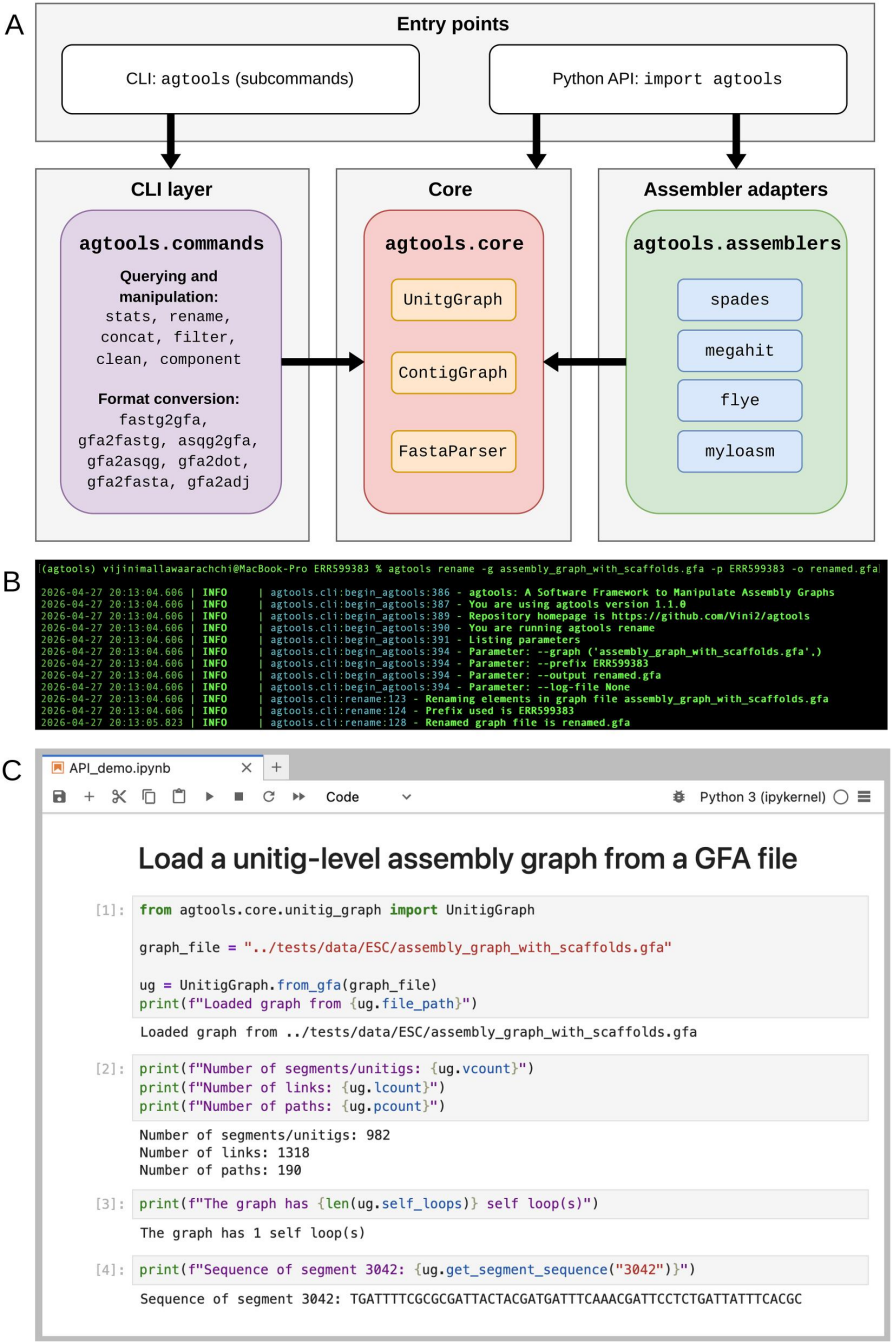
Overview of the system architecture and user interfaces of *agtools*. (A) The modular architecture of *agtools*, showing entry points, CLI layer, core graph abstractions, and assembler-specific adapters. Screenshots showing usage of the (B) CLI and (C) Python API.

The design of *agtools* follows a modular architecture, in which the core graph abstractions are separated from the CLI and assembler-specific logic, as illustrated in Fig. 1A. Core components such as the assembler-independent graph representations, sequence access, and related logic are separated into the agtools.core module. This module serves as the foundation for assembly-graph-based operations, enabling consistent behavior across both the API and CLI usage.

Built on top of this core, assembler-specific adapters in the agtools.assemblers module (e.g. SPAdes, MEGAHIT, Flye, and myloasm) translate tool-dependent assembly graphs into the common graph representation, abstracting away format differences and naming conventions. The CLI leverages these shared abstractions to provide utilities for inspection, transformation, and format conversion of assembly graphs. This modular organization promotes extensibility, simplifies maintenance, and allows graph representations from new assemblers, analyses or manipulation steps to be integrated without modifying the core logic.

### 2.4 Command line interface

*agtools* provides a CLI for users to analyze and manipulate assembly graphs. It includes subcommands for obtaining basic information, graph manipulation, and format conversion, as depicted in the CLI layer (under the agtools.commands module) in Fig. 1A. Figure 1B denotes a screenshot of an example run of the rename subcommand in the terminal. The CLI was developed using the Click package (https://click.palletsprojects.com/en/stable/).

### 2.5 Python package interface

*agtools* provides a Python package interface for users who want to represent and explore assembly graphs in their own bioinformatic tools and workflows. It provides an API to load graphs from GFA files. This is facilitated through the function from_gfa in the UnitigGraph class (in the agtools.core module in Fig. 1A), which loads and stores the graph as a python-igraph (Csárdi and Nepusz 2006) Graph object, allowing *agtools* to use igraph’s built-in graph functionality for various tasks such as traversing graphs, identifying connected components and obtaining neighbors of a given vertex. Unitig sequences (or segments) are stored using Biopython (Cock *et al*. 2009) Seq objects to easily obtain attributes such as length and reverse complements.

*agtools* allows users to load different assembly graphs from GFA files produced from four popular assemblers: SPAdes (Bankevich *et al*. 2012), MEGAHIT (Li *et al*. 2015), Flye (Kolmogorov *et al*. 2019) and myloasm (Shaw *et al*. 2026). Depending on the assembler, these graph representations may be obtained either directly from the GFA file or by incorporating contig path information associated with the assembly graph. Since SPAdes and Flye provide unitig-level assembly graphs and contigs resolved from the unitig graph, *agtools* provides two functions get_unitig_graph (built using the UnitigGraph class) and get_contig_graph (built using the ContigGraph class [in the agtools.core module in Fig. 1A]) to load the unitig graph and contig graph, respectively. MEGAHIT and myloasm produce contig-level assembly graphs with subtle differences in sequence representation which have been included in their respective get_contig_graph functions. If there are no assembler-specific intricacies and contig sequences are represented in the GFA files, assembly graphs from other assemblers can be loaded using the UnitigGraph class. Figure 1C shows a simple example of loading an assembly graph using the *agtools* API in a Jupyter Notebook. Further information about the API can be found in the online documentation.

### 2.6 Performance enhancements

*agtools* incorporates a number of performance optimizations. When storing sequences within the UnitigGraph and ContigGraph objects, it uses an integer index instead of the original sequence identifier when building the graph which facilitates faster access. Moreover, *agtools* stores file pointers to the lines that have the sequences instead of loading the full sequences into memory. The core functionality, including building indexes and retrieving sequences, is implemented in the FastaParser class (in the agtools.core module in Fig. 1A). Separate functions are implemented to retrieve sequences from the GFA file in the UnitigGraph class and from the FASTA file in the ContigGraph class when required. This approach significantly reduces memory usage during graph processing.

When concatenating multiple GFA files using the concat subcommand, *agtools* writes the different types of lines into separate temporary files and combines them at the end, rather than keeping all lines in memory. This approach is more memory efficient, especially when concatenating very large graph files with sizes on the order of gigabytes (refer to performance results of concat in Table 3, available as supplementary data at *Bioinformatics Advances* online).

## 3 Results and discussion

### 3.1 Performance analysis

We used short-read datasets from Tara Oceans (de Vargas *et al*. 2015) and long-read datasets from human gut metagenomes (Chen *et al*. 2022) and the Microflora Danica sequencing project (Sereika *et al*. 2025) to analyze the performance of *agtools*. The runs used from each study can be found in Table 1, available as supplementary data at *Bioinformatics Advances* online. We obtained assemblies from the following assembler versions: SPAdes v3.15.5 with the --meta flag (Nurk *et al*. 2017), MEGAHIT v1.2.9, Flye v2.9.6-b1802 with the --meta flag (Kolmogorov *et al*. 2020), and myloasm v0.1.0. All assemblers were run with default parameters.

We compared the performance of *agtools* (version 1.0.2) with GfaPy (version 1.2.3) (Gonnella and Kurtz 2017), which is the closest existing up-to-date Python-based tool in terms of functionality. For this comparison, we used the converted GFA files from MEGAHIT (converted using the fastg2gfa command) and GFA files from SPAdes and Flye. However, we could not run GfaPy on the myloasm assemblies due to format errors. The GFA files were loaded using the from_gfa function of the UnitigGraph class. The different contig graph representations of the *agtools* API were also profiled. The running time was recorded using cProfile (https://docs.python.org/3/library/profile.html), and memory usage was recorded using pympler (https://github.com/pympler/pympler). Each dataset was run 10 times and the minimum, maximum, mean and standard deviation of the running time (the wall clock time between start and finish) were recorded. The CLI commands were profiled using the/usr/bin/time command. Each command was run 10 times on each dataset. The elapsed (wall clock) time and peak memory usage (maximum resident set size) were recorded. All profiling jobs were run on a single AMD EPYC 7742 @2.25 GHz CPU node provided by the Flinders University’s DeepThought high-performance computing (HPC) platform (Flinders University 2021). All performance analysis results can be found in the Supplementary Material, available as supplementary data at *Bioinformatics Advances* online.

**Figure 2:**
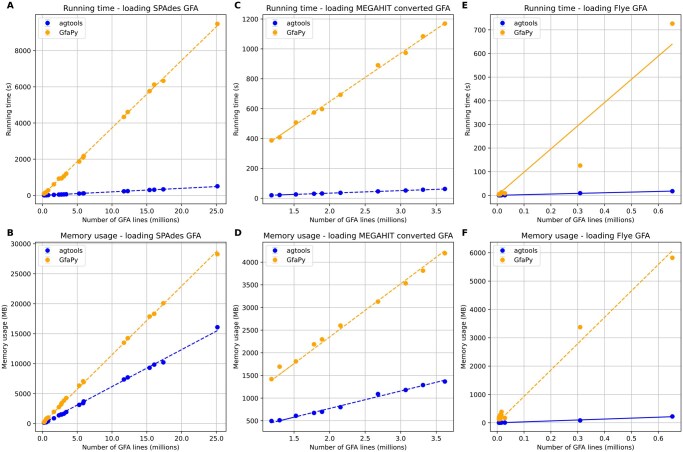
Performance analysis results of *agtools* and GfaPy for loading GFA files. Running time (A, C, E) and memory usage (B, D, F) for SPAdes GFA files of the Tara Oceans assemblies (A, B), converted GFA files of MEGAHIT of the Tara Oceans assemblies (C, D) and Flye GFA files of the human gut metagenomes and the Microflora Danica assemblies (E, F). The x axis denotes the sum of the number of S (segments), L (links), and P (paths) lines in the GFA file (in millions).

Figure 2 shows that *agtools* consistently outperforms GfaPy in both runtime and memory usage across assembly graphs from different assemblers. The resource usage for loading graphs scales linearly with respect to the number of S (segments), L (links), and P (paths) lines in the GFA file. This indicates that the computational cost for graph loading grows in proportion to the size of the input graph representation. The performance results for loading contig graphs follow similar patterns as unitig graphs and can be found in Figs 1–4, available as supplementary data at *Bioinformatics Advances* online.

Although parsing large GFA files can take several minutes, this reflects the cost of constructing a fully indexed in-memory graph representation rather than simple file parsing. Importantly, the runtime scales approximately linearly with the number of GFA lines, indicating predictable performance for large assemblies. Compared to GfaPy, *agtools* achieves substantially lower runtime and memory usage while providing additional functionality for downstream graph-based analysis. This performance difference is in part due to the design choices in *agtools* that assume input files conform to the GFA specifications and, therefore, perform less extensive validation than tools such as GfaPy.

### 3.2 Example applications

The following example applications illustrate how *agtools* can be used to extract and manipulate assembly graphs for downstream analyses. These examples are intended to demonstrate typical usage patterns rather than provide complete implementations of the respective analytical methods. Example code for the following use cases is available in the online documentation.

#### 3.2.1 Metagenomic binning

Contigs connected together in the assembly graph are more likely to belong to the same taxonomic group (Mallawaarachchi *et al*. 2020a, 2020b, 2021, Mallawaarachchi and Lin 2022a). *agtools* can be used to obtain connected components in the contig-level assembly graph, allowing users to group contigs into candidate bins that represent different taxonomic groups. These candidate bins can be further refined in later steps by either splitting when multiple species are in one component (e.g. using single-copy marker genes; Mallawaarachchi and Lin 2022a) or merging when multiple components belong to the same species.

#### 3.2.2 Identifying plasmid candidates

Plasmids are often assembled as circular segments in the assembly (Bouras *et al*. 2023). Hence, identifying circular segments within the typical range of plasmid genome lengths is a good starting point before proceeding to deeper validation to identify plasmids. *agtools* can be used to identify such circular segments using self-loops (i.e. an edge that connects a node to itself) that are not connected to any other segment in the assembly graph.

#### 3.2.3 Identifying candidate bacteriophage genomes

Bacteriophages with circular genomes can form circular components in the assembly graph (Mallawaarachchi *et al*. 2023). Hence, we can identify bacteriophage candidates by determining components that have circular genomic paths. *agtools* can be used to traverse the paths within components of the assembly graph. These paths can be filtered based on the typical range of bacteriophage genome lengths before further validation, such as scanning for highly conserved bacteriophage genes (Mallawaarachchi *et al*. 2023).

#### 3.2.4 Haplotype phasing

Haplotype phasing is the process of identifying and reconstructing genetic variants (alleles) of haplotypes in diploid or polyploid genomes. This can be achieved by resolving bubbles in assembly graphs where a vertex splits into multiple outgoing edges that later re-converge into another vertex (Weisenfeld *et al*. 2017, Garg *et al*. 2018). These alternative paths in the graph correspond to sequence differences between haplotypes. *agtools* can be used to parse the assembly graph and traverse paths to identify bubbles.

## 4 Conclusion

*agtools* is an open-source Python framework designed to support programmatic access to analyze and manipulate assembly graphs. It focuses on providing a standardized, extensible, and reproducible software layer for assembly-graph-based bioinformatics analyses. By exposing both a CLI and a Python package interface, *agtools* enables users to integrate assembly graph operations into custom workflows, prototype new analysis methods, and develop downstream tools without re-implementing format-specific logic.*agtools* is implemented using a modular architecture that allows individual components to be reused. It can be easily extended to add more functionality, enabling the addition of new graph operations and support for new formats. In future work, we plan to incorporate more comprehensive GFA input validation, add support for more assemblers that generate assembly graphs, and integrate more features such as graph-based sequence querying, which will broaden the utility of *agtools* in other genomic and metagenomic applications.

## Supplementary Material

vbag126_Supplementary_Data

## Data Availability

All datasets containing raw sequencing data used for this work are publicly available from their respective studies on NCBI. The Tara Oceans datasets were downloaded from BioProject number PRJEB4419, the human gut metagenomes from PRJNA820119, and Microflora Danica datasets from PRJEB58634. The assembly outputs are available on Zenodo at https://zenodo.org/records/17075231 (short-read assemblies) and https://zenodo.org/records/17096649 (long-read assemblies).

## Code availability

The code of *agtools* and all the performance analysis results are available on GitHub at https://github.com/Vini2/agtools under the MIT license.
