# Supplementary material for: agtools: a software framework to manipulate assembly graphs
Source: Bioinform Adv. 2026 May 5;6(1):vbag126. doi: 10.1093/bioadv/vbag126 (PMC13197128; doi:10.1093/bioadv/vbag126)
Supplement: vbag126_Supplementary_Data [file vbag126_supplementary_data.pdf]

agtools: a software framework to manipulate assembly graphs

Supplementary Material

Vijini Mallawaarachchi[1*], George Bouras[2,3], Ryan R. Wick[4,5], Susanna R. Grigson[1,6], Bhavya Papudeshi[1,7], Robert A. Edwards[1]

[1] Flinders Accelerator for Microbiome Exploration, College of Science and Engineering, Flinders University, Bedford Park, Adelaide, SA 5042, Australia

[2] School of Medicine, College of Health, Adelaide University, Adelaide, SA 5005, Australia.

[3] The Department of Surgery – Otolaryngology Head and Neck Surgery, Central Adelaide Local Health Network, Adelaide, SA 5005, Australia.

[4] Department of Microbiology and Immunology, The University of Melbourne at the Peter Doherty Institute for Infection and Immunity, Melbourne, VIC 3000, Australia

[5] Centre for Pathogen Genomics, The University of Melbourne, Parkville, VIC 3010, Australia

[6] DOE Joint Genome Institute, Lawrence Berkeley National Laboratory, Berkeley, CA 94720, United States.

[7] Department of Fundamental Microbiology, University of Lausanne, Lausanne, CH-1005, Switzerland.

* Corresponding author

Runs used for performance analysis

Table S1. Datasets used for profiling *agtools*

Study	Bioproject number	SRA accession numbers used in this work
Tara Oceans (de Vargas et al. 2015)	PRJEB4419	ERR2750826, ERR2750828, ERR2752143, ERR2752144, ERR2752145, ERR2752146, ERR2752147, ERR2752149, ERR2752150, ERR2752151, ERR2752153, ERR2752154, ERR2752160, ERR2752162, ERR2752163, ERR321018, ERR594355, ERR594360, ERR594361, ERR594362, ERR594370, ERR594371, ERR594372, ERR594375, ERR599357, ERR599362, ERR599370, ERR599383
Human gut (Chen et al. 2022)	PRJNA820119	SRR18490951, SRR18490961, SRR18491036, SRR18491148, SRR18491176, SRR18491204, SRR18491300, SRR18491309, SRR18491312, SRR18491319
Microflora Danica (Sereika et al. 2025)	PRJEB58634	ERR12040030, ERR11593880, ERR11561019, ERR11523645, ERR10750395

Running time and memory usage of `rename`

Table S2. Running time and memory usage of `rename` subcommand on the Tara Oceans datasets.

Dataset	Size of FASTG file	Wall-clock time (seconds)				Peak memory usage (GB)			
		Mean	Min	Max	STD	Mean	Min	Max	STD
ERR2752151	2.6GB	55.73	54.77	57.46	0.76	2.549	2.549	2.549	1.21×10^{-4}
ERR2752163	2.5GB	43.14	41.07	44.74	1.31	1.616	1.616	1.616	3.73×10^{-5}
ERR2752162	2.2GB	38.43	36.56	41.23	1.43	1.473	1.472	1.474	3.98×10^{-4}
ERR2752160	2.0GB	38.99	37.59	40.79	0.82	1.477	1.476	1.478	4.99×10^{-4}
ERR2752150	1.7GB	29.45	28.37	30.16	0.62	1.327	1.327	1.327	1.36×10^{-5}
ERR2752154	1.5GB	27.88	26.66	28.36	0.51	1.326	1.326	1.326	3.14×10^{-5}
ERR2752145	1.4GB	23.53	21.84	25.93	1.06	0.916	0.916	0.916	0
ERR2752149	1.4GB	23.53	22.77	24.10	0.50	0.903	0.903	0.904	8.93×10^{-5}
ERR2752152	1.1GB	18.45	17.99	19.78	0.54	0.770	0.770	0.770	1.71×10^{-4}
ERR2752153	1.2GB	15.79	15.58	16.04	0.15	0.716	0.716	0.717	1.05×10^{-4}

Running time and memory usage of `concat`

Table S3. Running time and memory usage of `concat` subcommand on the Tara Oceans datasets. Multiple datasets were concatenated using the `concat` subcommand.

Number of GFA files concate-nated	Total size of GFA files (GB)	Wall-clock time				Peak memory usage (GB)			
		Mean	Min	Max	STD	Mean	Min	Max	STD
2	5.8	55.9s	34.9s	1m 9.4s	10.1s	2.075	1.022	3.112	0.629
3	8.2	1m 18.7s	1m 10.2s	1m 33.2s	8.3s	3.017	2.086	3.165	0.328
4	9.3	2m 0.4s	1m 41.2s	2m 10.6s	9.2s	3.704	3.252	3.843	0.205
5	10.5	2m 33.1s	2m 22.7s	3m 0.2s	10.5s	6.121	6.120	6.123	6.61×10^{-4}
6	12.4	2m 47.1s	2m 40.9s	2m 50.9s	3.4s	6.121	6.121	6.123	8.23×10^{-4}
7	14.1	3m 3.9s	2m 53.9s	3m 27.1s	9.6s	6.121	6.121	6.123	7.83×10^{-4}
8	15.3	3m 24.5s	3m 7.4s	4m 31.8s	24.6s	6.196	6.195	6.199	1.45×10^{-3}
9	16.3	3m 19s	3m 14.4s	3m 25.6s	3.5s	6.423	6.421	6.425	1.10×10^{-3}
10	17.2	3m 29.2s	3m 22s	3m 34.7s	4.4s	6.571	6.569	6.574	1.51×10^{-3}
11	18.0	3m 45s	3m 39.2s	3m 50.5s	4.5s	6.934	6.932	6.937	1.62×10^{-3}

Running time and memory usage of `clean`

Table S4. Running time and memory usage of `clean` subcommand on the Microflora Danica datasets.

Dataset	Size of GFA file (GB)	Size of FASTA file (GB)	Wall-clock time				Peak memory usage (GB)			
			Mean	Min	Max	STD	Mean	Min	Max	STD
ERR12040030	0.940	25	4m 58s	4m 52s	5m 3s	3.5s	0.960	0.959	0.960	1.88×10^{-4}
ERR11593880	0.782	22	4m 28s	4m 13s	5m 28s	22.6s	0.722	0.721	0.723	6.36×10^{-4}
ERR11523645	1.004	12	2m 36s	2m 29s	2m 50s	6.8s	0.544	0.543	0.545	2.97×10^{-4}
ERR11561019	0.286	6.5	1m 28s	1m 24s	1m 36s	4.2s	0.343	0.343	0.343	8.87×10^{-5}
ERR10750395	0.047	0.416	12.6s	12.1s	13.5s	0.4s	0.139	0.139	0.140	8.16×10^{-5}

Running time and memory usage of `fastg2gfa`

Table S5. Running time and memory usage of `fastg2gfa` subcommand on the Tara Oceans datasets.

Dataset	Size of FASTG file	Wall-clock time (seconds)				Peak memory usage (GB)			
		Mean	Min	Max	STD	Mean	Min	Max	STD
ERR2752163	3.4GB	19.5	18.2	23.8	1.7	6.325	6.323	6.327	6.04×10^{-4}
ERR2752147	3.1GB	17.7	16.7	22.2	1.6	5.707	5.706	5.707	5.07×10^{-4}
ERR2752151	2.8GB	16.1	15.2	21.1	1.8	5.264	5.264	5.265	2.89×10^{-5}
ERR2752146	2.5GB	15.1	13.5	24.6	3.3	4.729	4.728	4.729	1.04×10^{-4}
ERR2752150	2.3GB	12.5	11.5	15.3	1.1	4.104	4.103	4.104	3.01×10^{-4}
ERR2752145	2.0GB	11.7	10.3	20.5	3.1	3.649	3.648	3.650	6.31×10^{-4}
ERR2752149	1.9GB	10.9	10.3	13.0	0.8	3.526	3.526	3.528	7.88×10^{-4}
ERR2752153	1.6GB	8.8	8.5	10.0	0.4	2.867	2.867	2.867	2.95×10^{-5}
ERR2752144	1.5GB	8.7	8.5	9.1	0.2	2.833	2.832	2.833	1.69×10^{-5}
ERR2752143	1.2GB	7.2	6.8	9.1	0.7	2.233	2.233	2.234	2.68×10^{-5}

cProfile profiling results on contig-level assembly graphs

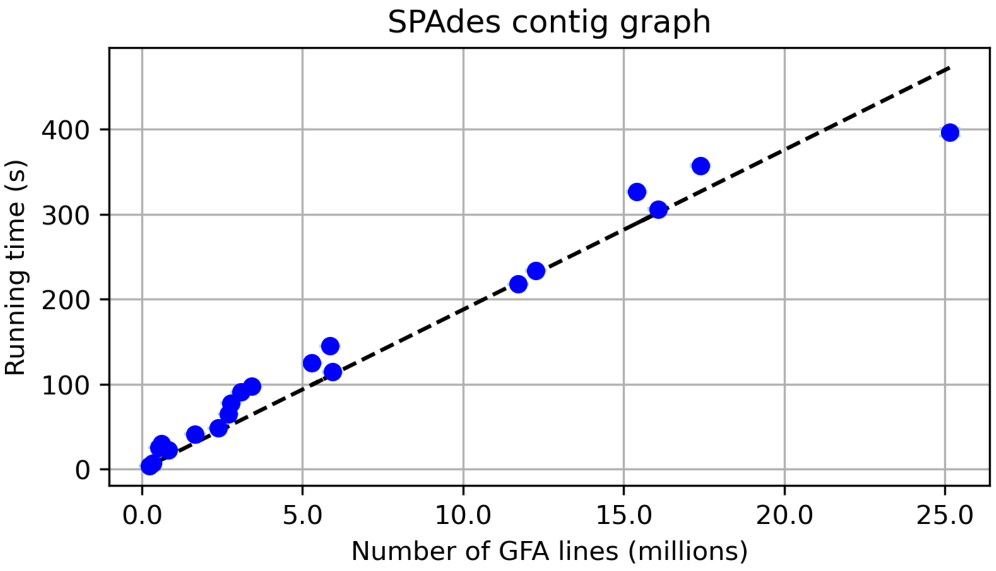

Figure S1. Running time to load contig-level assembly graphs from SPAdes assemblies of the Tara Oceans datasets.

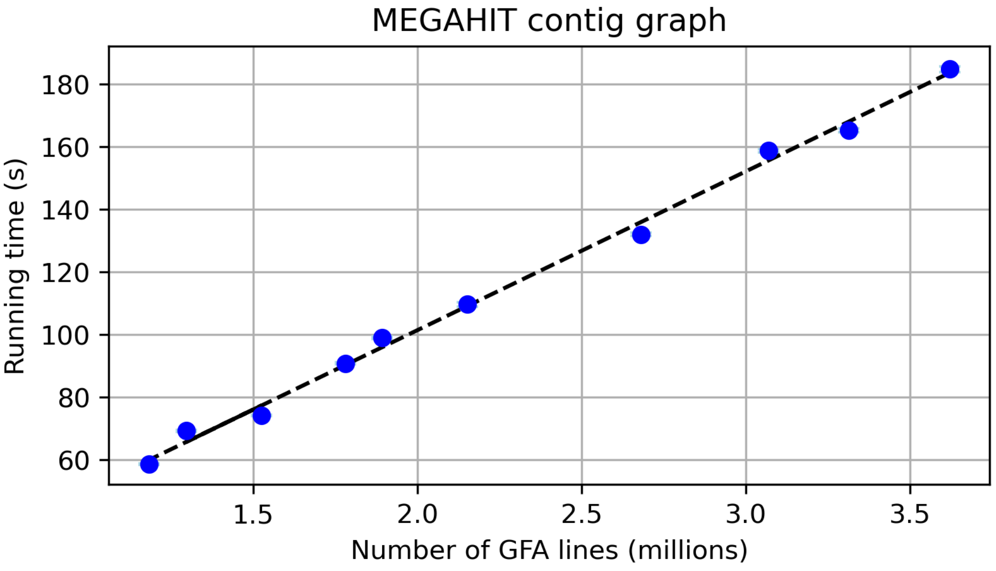

Figure S2. Running time to load contig-level assembly graphs from MEGAHIT assemblies of the Tara Oceans datasets.

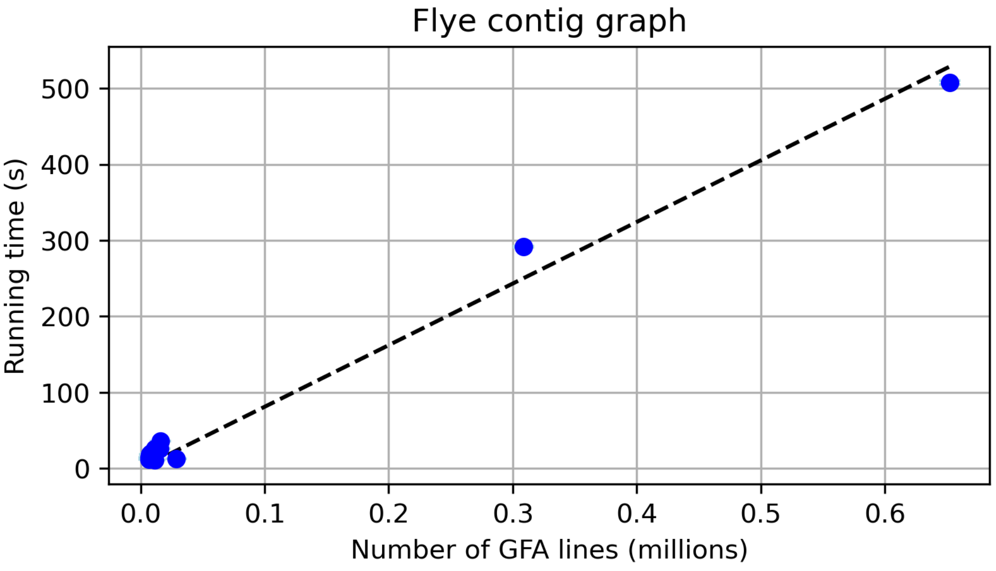

Figure S3. Running time to load contig-level assembly graphs from Flye assemblies of the Human gut metagenome and Microflora Danica datasets.

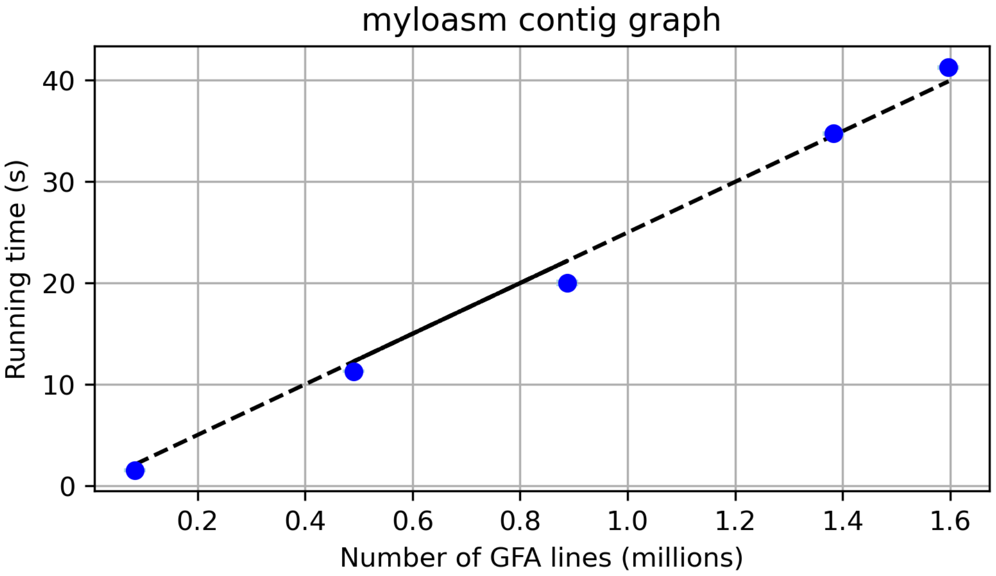

Figure S4. Running time to load contig-level assembly graphs from myloasm assemblies of the Microflora Danica datasets.

Assemblers used

1. metaSPAdes (Nurk et al. 2017) from SPAdes (Bankevich et al. 2012)
2. MEGAHIT (Li et al. 2015)
3. metaFlye (Kolmogorov et al. 2020) from Flye (Kolmogorov et al. 2019)
4. myloasm (Shaw et al. 2025)

Raw results

All raw data for the tables above, along with the code used to generate the figures, are available in the *agtools* GitHub repository at https://github.com/Vini2/agtools/tree/main/profiling.

References

Bankevich, Anton, Sergey Nurk, Dmitry Antipov, et al. 2012. *SPAdes: A New Genome Assembly Algorithm and Its Applications to Single-Cell Sequencing*. May 7.

Chen, Liang, Na Zhao, Jiabao Cao, et al. 2022. “Short- and Long-Read Metagenomics Expand Individualized Structural Variations in Gut Microbiomes.” *Nature Communications* 13 (1): 3175.

Kolmogorov, Mikhail, Derek M. Bickhart, Bahar Behsaz, et al. 2020. “metaFlye: Scalable Long-Read Metagenome Assembly Using Repeat Graphs.” *Nature Methods* 17 (11): 1103–1110.

Kolmogorov, Mikhail, Jeffrey Yuan, Yu Lin, and Pavel A. Pevzner. 2019. “Assembly of Long, Error-Prone Reads Using Repeat Graphs.” *Nature Biotechnology* 37 (5): 540–546.

Li, Dinghua, Chi-Man Liu, Ruibang Luo, Kunihiko Sadakane, and Tak-Wah Lam. 2015. “MEGAHIT: An Ultra-Fast Single-Node Solution for Large and Complex Metagenomics Assembly via Succinct de Bruijn Graph.” *Bioinformatics (Oxford, England)* 31 (10): 1674–1676.

Nurk, Sergey, Dmitry Meleshko, Anton Korobeynikov, and Pavel A. Pevzner. 2017. “metaSPAdes: A New Versatile Metagenomic Assembler.” *Genome Research* 27 (5): 824–834.

Sereika, Mantas, Aaron James Mussig, Chenjing Jiang, et al. 2025. “Genome-Resolved Long-Read Sequencing Expands Known Microbial Diversity across Terrestrial Habitats.” *Nature Microbiology* 10 (8): 2018–2030.

Shaw, Jim, Maximillian G. Marin, and Heng Li. 2025. “High-Resolution Metagenome Assembly for Modern Long Reads with Myloasm.” In *bioRxiv*. September 6.

Vargas, Colomban de, Stéphane Audic, Nicolas Henry, et al. 2015. “Ocean Plankton. Eukaryotic Plankton Diversity in the Sunlit Ocean.” *Science (New York, N.Y.)* 348 (6237): 1261605.
